# LLM-Enhanced multimodal detection of fake news

**DOI:** 10.1371/journal.pone.0312240

**Published:** 2024-10-24

**Authors:** Jingwei Wang, Ziyue Zhu, Chunxiao Liu, Rong Li, Xin Wu

**Affiliations:** 1 School of Humanities and Communication, Zhejiang Gongshang University, Hangzhou, China; 2 School of Information and Electronic Engineering, Zhejiang Gongshang University, Hangzhou, China; 3 School of Computer Science and Technology, Zhejiang Gongshang University, Hangzhou, China; University of Sargodha, PAKISTAN

## Abstract

Fake news detection is growing in importance as a key topic in the information age. However, most current methods rely on pre-trained small language models (SLMs), which face significant limitations in processing news content that requires specialized knowledge, thereby constraining the efficiency of fake news detection. To address these limitations, we propose the FND-LLM Framework, which effectively combines SLMs and LLMs to enhance their complementary strengths and explore the capabilities of LLMs in multimodal fake news detection. The FND-LLM framework integrates the textual feature branch, the visual semantic branch, the visual tampering branch, the co-attention network, the cross-modal feature branch and the large language model branch. The textual feature branch and visual semantic branch are responsible for extracting the textual and visual information of the news content, respectively, while the co-attention network is used to refine the interrelationship between the textual and visual information. The visual tampering branch is responsible for extracting news image tampering features. The cross-modal feature branch enhances inter-modal complementarity through the CLIP model, while the large language model branch utilizes the inference capability of LLMs to provide auxiliary explanation for the detection process. Our experimental results indicate that the FND-LLM framework outperforms existing models, achieving improvements of 0.7%, 6.8% and 1.3% improvements in overall accuracy on Weibo, Gossipcop, and Politifact, respectively.

## Introduction

With the advancement of media technology and shifts in social ecology, coupled with the deep penetration and extensive utilization of social media platforms globally, the production and dissemination of information have reached unprecedented levels. However, in this flood of information, fake news maliciously misleads public perceptions, causing significant negative social impacts. The term “fake news” denotes news that is intentionally fabricated and verifiably false [1, 2]. Often cloaked in the guise of authentic news, these news contain conspicuous falsehoods that seriously infringe upon the public’s right to be informed and significantly compromise the health of the information ecosystem. The proliferation of fake news challenges the authority of traditional media and triggers serious consequences in areas including political elections economic decisions,and pubic society [3]. It readily incites public emotions, exacerbates social divisions, provokes economic turmoil, and even jeopardize public safety during critical moments.

To curb the proliferation of fake news, manual verification methods have proven effective, but they are costly, laborious, and time-consuming, and unable to cope with today’s exponential growth of information data. Consequently, automated detection of fake news has become an issue of great concern in recent years [2, 4, 5]. Early fake news detection works focused on text-only [6] or image-only [7] content analysis, without fully considering the potential correlation between the two. For example, in some fake news cases, real images may be accompanied by false textual content, or correct textual content is used to describe doctored images. Therefore, multimodal feature analysis is necessary to provide complementary advantages for fake news detection. In recent years, multimodal fake news detection has gradually become a research hotspot and has made significant progress [8, 9]. However, fake news often exhibits complex structures and content-related characteristics, requiring a deep understanding of domain-specific knowledge for accurate evaluation. Certain fake news items necessitate expertise in specific fields to be effectively identified [10]. Currently, research methods for fake news detection commonly utilize models such as BERT [11] and RoBERTa [12] and RoBERTa to understand news content and extract its underlying representations. While SLM do offer some improvements, the limitations in their knowledge and processing capabilities also restrict further enhancements in the performance of fake news detection. For example, since BERT is pre-trained on text corpora such as Wikipedia [11], it has difficulty processing news items that require specific knowledge but are not included in the training set [13].

Wei et al. [14] and Chen et al. [15] emphasizes the importance of cross-modal analysis, exploiting cross-modal feature correlations to construct models for fusing images and text. However, this paper recognizes that cross-modal features are not a decisive factor in all cases. In some cases, unimodal content is sufficient to assess the credibility of a news, whereas models that overly rely on multimodal fusion may lead to misclassification due to feature mismatches. Therefore, clarifying the roles of unimodal and cross-modal features is crucial to improve the efficiency of fake news detection.

Furthermore, user features [16], Social network information [17], dissemination structures [18] and knowledge graphs [19, 20] and other factors have also been proven to possess potential value for fake news detection. However, a few studies have revealed that relying solely on user features is insufficient to detect fake news [21]. On the one hand, the comments written by individuals are often influenced by subjective emotions and positions, and it is difficult to achieve absolute objectivity and fairness; On the other hand, certain users intentionally manipulate public opinion, fostering a herd mentality among ordinary users and resulting in a proliferation of irrational comments. These comments are not only of limited help in identifying the fake news, but may even confuse the public and intensify the spread of misinformation.

Combining the above issues, this paper finds that there are still some challenges in multimodal fake news detection methods:

- Pre-trained Small Language Model (SLM) such as BERT and RoBERTa have limited capability in processing specific knowledge, which restricts the efficiency of fake news detection.
- While multimodal fake news detection provides complementary advantages, over-reliance on modal fusion in research may lead to misclassification due to cross-modal feature conflicts or weak correlations.
- User features hold potential value for fake news detection, but social context information from user interactions is too heterogeneous and of varying quality to be used to directly assess the credibility of news content.

In order to address the above challenges, this paper proposes a LLM-Enhanced multimodal fake news detection method (FND-LLM). In this technical framework, a deep learning method is used which contains different branches specialized in processing text and image information. For images, VIT(Vision Transformer) and CLIP are used to extract visual semantic information, and visual tampering features are extracted using Extended Autocoded ViT (EAViT); for text, BERT and CLIP are used to encode text. The outputs of the text and image branches are then fed into a Co-attention Transformer network, where CLIP is responsible for detecting similarities between text and images and integrating them into a multimodal space. In addition, a Rationales Encoder is included, which interacts with the LLMs to provide logical reasoning and external validation. Ultimately, the multidimensional features are further integrated via the MMoE network, and a classifier is then used to differentiate between fake and real news.

The main contributions of this paper are:

- We propose a novel method to fake news detection that combines SLMs and LLMs to complement each other. This method leverages the large language models to generate explanatory justifications, providing guiding suggestions from multiple perspectives for multimodal fake news detection.
- We design unimodal branches for text and images, and adopt a CLIP pre-training model to evaluate cross-modal correlations. Furthermore, the separation and adaptive tuning of unimodal and multimodal features are achieved through uniview prediction and cross-modal consistency learning, thereby optimizing the detection results.
- We validate our method on three real-world news datasets in both Chinese and English. Compared to existing baseline methods, the FND-LLM significantly enhances detection accuracy and the precision of fake news identification.

## Related work

### Unimodal-based fake news detection

Unimodal fake news detection can be categorized into two types: text feature-based methods and visual feature-based methods. Early fake news detection efforts focused on news textual features to detect the authenticity of articles by analyzing post text, user profiles, social metadata and retweets [22]. Most of these textual features are created manually [23, 24], which not only is time-consuming but also hampers the full exploration of the deep semantic information conveyed by the text. To overcome the shortcomings of manual feature extraction, many researchers utilize deep learning techniques to extract more comprehensive and generalized news features. Ma et al. [25] sequentially process each time step of rumor propagation based on recurrent neural network (RNN); Chen et al. [26] improve the RNN through the attention mechanism; Liao et al. [27] propose a graph-based method for learning news representations that capture news relationships.

Visual features have also been shown to be used as single modal features to identify fake news. Jin et al. [28] characterize the distribution patterns of images by extracting multiple visual features. Cao et al. [29] discover that typical image processing detection methods [30] help to reveal traces of fake news image tampering. Qi et al. [31] design a convolutional neural network (CNN)-based model for capturing the complex patterns of fake news images in the frequency domain and use a multi-branch CNN-RNN model to extract visual features from different semantic levels in the pixel domain, and finally dynamically fuse feature representations of the two domains using an attention mechanism.

### Multimodal-based fake news detection

In the field of fake news detection, multimodal methods show strong potential and advantages due to their ability to synthesize text, image and other modal information. In recent years, with the rapid development of natural language processing and computer vision technologies, more and more studies have begun to explore how to effectively combine these modalities for fake news detection. Vaswani et al. [32] introduce the Transformer model, which significantly improves the ability to process sequential data through the self-attention mechanism, and provides strong theoretical support and technical foundation for subsequent cross-modal tasks. Jin et al. [33] address the problem of fake news detection for the first time by combining deep neural networks with multimodal content. Their study proposes an innovative RNN with attention mechanism (attRNN) that can effectively fuse textual, visual and social contextual features. Singhal et al. [34] propose the SpotFake model, which uniquely combines the processing of textual and visual information by embedding textual content using the BERT algorithm and embedding image content using the VGG19 network, which enables in-depth learning of contextual information from the input data. Wang et al. [35] propose an Event Adversarial Neural Network (EANN) model to better capture the generic features of fake news by fusing text and image content and employing an adversarial training strategy, and also utilizing an event discriminator to remove event-specific features and retain shared features between events. Khattar et al. [36] reconstruct the text and image content of news using Multimodal Variational Auto-Encoder (MVAE) to efficiently extract and fuse features between different modalities. Ma et al. [37] utilized graph models to extract more advanced representations of propagation paths from disseminated information. Lao et al. [38] employed spectral methods for multimodal feature fusion representation.

In addition, some methods utilize cross-modal semantic associations for fake news detection. Xue et al. [5] propose a detection method that explores multimodal data consistency (MCNN), combining textual semantic features, visual tampering features, and similarity between textual and visual information for detecting fake news. However, the aggregation process merely concatenates all the features, making it impossible to measure the impact of these multidimensional features on prediction. Zhou et al. [39] develop the SAFE model, which distinguishes fake news by measuring the intra-modal relationship within modalities and the cross-modal similarity of the news. Chen et al. [15] propose a cross-modal ambiguity learning (CAFE) method to detect fake news by analyzing and learning potential ambiguities between different modalities such as text and images. However, when dealing with news that have high consistency between graphical and textual content, the CAFE method may over-emphasize the inter-modal consistency and neglect the important information that may be carried within each individual modality, leading to misjudgment of certain news. These methods often rely on simple feature splicing or direct cross-mode similarity calculation, failing to fully consider the asymmetry of information between modes and its potential conflicts. The FND-LLM framework proposed in this paper adopts a co-attention network in the multimodal fusion process to effectively capture the complex semantic associations between text and images through cross-modal feature interaction and fusion.

Pre-trained language models (e.g., BERT and BART) are now widely used for fake news-related tasks and show excellent results. For example, Lee et al. [40] explore the possibility of utilizing a pre-trained language model, BERT, for fact-checking. Their study shows that BERT can be effectively used for fact-checking tasks without the need for an external knowledge base or explicit retrieval components, providing new ideas for using language models to verify the truthfulness of information. Lee et al. [41] adopt LLMs, such as GPT-2, to perform fact-checking through perplexity. With pre-trained LLMs, such as GPT-3.5, which boasts a large number of parameters, impressive capabilities are emerging in various downstream tasks [42, 43]. Consequently, LLMs are expected to become general-purpose task solvers [44]. Yao et al. [45] and Jiang et al. [46] explore how collaborative reasoning and behavior in language models to improve the model’s decision making and execution in complex tasks. Zhang et al. [47] implement a large language model (LLM)-based news fact verification by using a hierarchical step-by-step cueing method. This method effectively utilizes the capability of LLMs in processing complex textual information through decomposition and step-by-step guidance, revealing the potential of LLMs in the task of fake news detection. However, there is still limited research on utilizing the reasoning power of LLMs in the field of fake news. Different from Pelrine et al. [48] simply guided LLM prediction, this paper creatively LLM and the advantages of small language model, through LLM powerful reasoning ability generate explanatory reason, for the small model with rich background knowledge and context understanding, greatly enhance the overall performance of the model.

## Method

**Fig 1:**
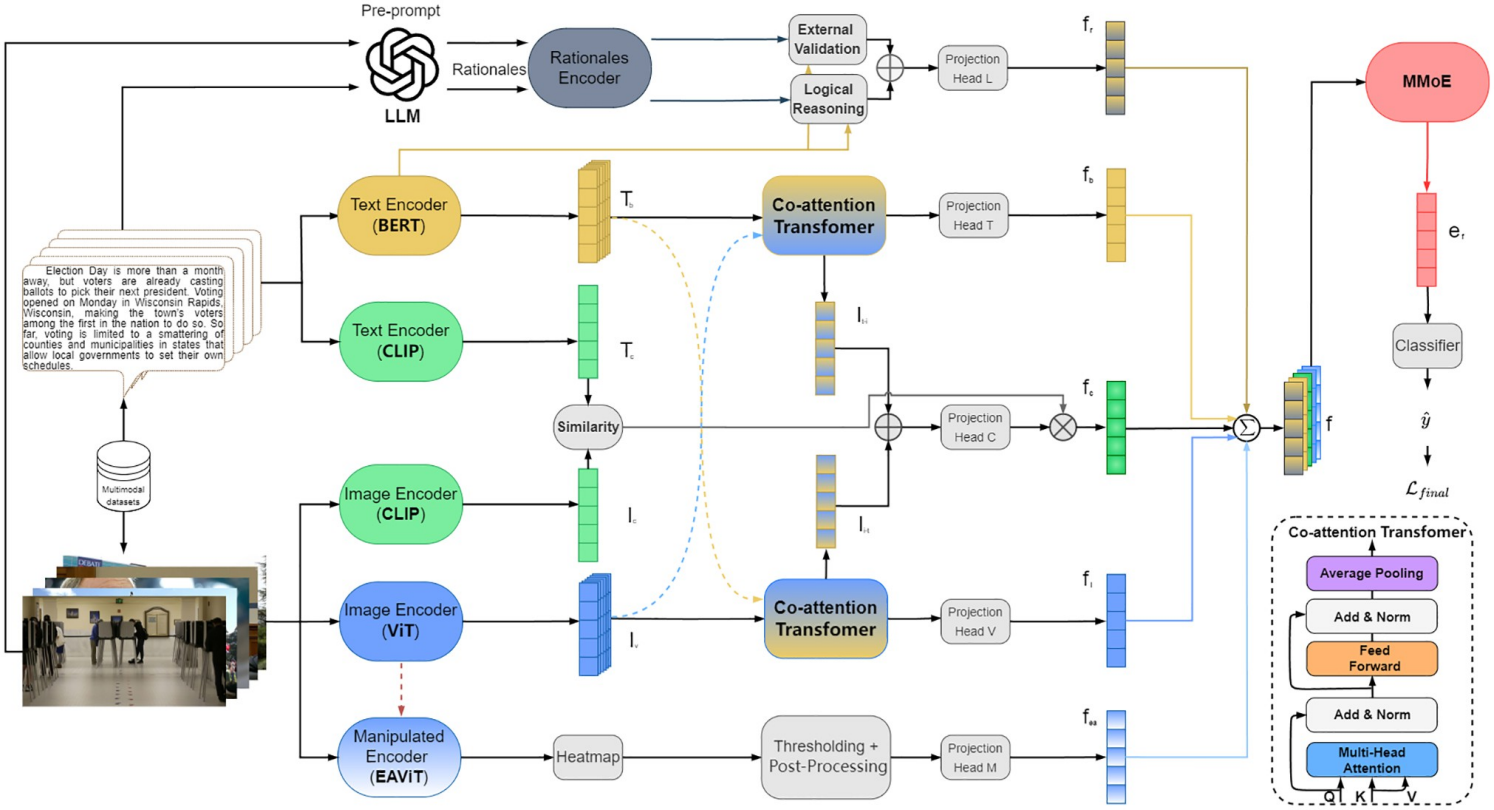
The FND-LLM model architecture.

In this paper, we propose a LLM-Enhanced multimodal fake news detection method (FND-LLM). It consists of eight modules, textual feature branch, visual semantic branch, visual tampering branch, co-attention network, cross-modal feature branch, large language model branch, multidimensional feature integration network and final classifier. The overall model architecture is shown in Fig 1. The symbols used in this paper are shown in Table 1.

**Table 1:** Abstract description.

Notation	Description
*T*	Input text sequence
**T** ^*b*^	Textual feature extracted using BERT model
*I*	Input image
**I** ^*c*^	Image feature vector extracted by CLIP Image Encoder
*P*	Sequence of image patches
*p*_*i*_	Individual image patch
**Z** ^(0)^	Initial sequence of projected image patches
**Z** ^(*l*)^	Output of the *l*-th layer in ViT
**Z** ^(*L*)^	Final output of the ViT encoder
*T*_*i*_	Image token obtained from image patch *p*_*i*_
*I*_*r*_	Reconstructed image after tampering detection
*f*_*L*_	Laplacian filter applied to input image
*Q*, *K*, *V*	Query, Key, and Value matrices in co-attention mechanism
*W*_*Q*_, *W*_*K*_, *W*_*V*_	Learnable transformation matrices in co-attention mechanism
*S*	Cosine similarity between image and text features
*F*	Fused feature vector
*F*_total_	Final fused feature representation for classification
**H**_*x*_	Hidden representation of news article encoded by BERT
*R*	Rationale generated by large language models (LLMs)
*E*	Integrated rationale insight vector from LLMs
*w*_*i*_	Weight assigned to each rationale embedding from LLMs
*L*(*u*)	Logical consistency score for logical units in text
score(*s*, *f*)	Similarity score between news statements and factual database
*g*_*k*_	Gating weights for expert networks in MMoE module
**F**_*k*_	Output feature vector from the *k*-th expert network
**y**	Predicted label (probability distribution)

### Textual feature branch

Textual features play an important role in text analysis and directly affect the accuracy of fake news detection.The textual feature branch is particularly adept at handling complex linguistic phenomena, such as puns, metaphors, or sarcasm, which frequently appear in fake news. BERT (Bidirectional Encoder Representations from Transformers) [11] is a popular pre-trained language model built on Transformer that uses the BERT model to encode features from *T*. The textual content of a news is a list of serialized words extracted and spliced from the text and images by optical character recognition (OCR), denoted as $T=[t_{1},t_{2},\ldots ,t_{n_{w}}]$, where is the number of words. After applying BERT to *T*, the encoded textual feature $T^{b}=\left[{t_{1}}^{b},{t_{2}}^{b},\ldots ,{t_{n}}^{b}\right]$ is obtained, where ${t_{i}}^{b}\in R^{d_{b}}$ is the output of the last hidden state of the *i*th text unit in the text embedding and *d*_*b*_ is the dimension of the word embedding. The text encoder is shown in Eq (1):

$$\begin{array}{c} T^{b}={\text{BERT}}_{Encoder}\left(T\right) \end{array}$$ (1)

where, *BERT*_Encoder_ represents the encoding function of the BERT model that converts the input text sequence *T* into a sequence of embedding vectors **T**^*b*^. For each word *T*_*i*_ the BERT output $t_{i}^{b}$, selects the hidden state of the last layer of BERT, which represents the high-level features of the context considered in the BERT model.

### Visual semantic branch

Vision Transformer (ViT) is a Transformer model applied directly to image pixels, aiming at capturing fine-grained semantic information in images. The visual semantic branch captures visual features that not only provide critical supplementary information for fake news detection but also assist in identifying misleading content conveyed through manipulated images. It translates traditional natural language processing techniques applied to the visual domain by segmenting an image into a series of image blocks and then serializing these blocks. Unlike traditional Convolutional Neural Networks (CNNs), Vision Transformer (ViT) excels at capturing global semantic relationships. ViT leverages a self-attention mechanism to better handle long-range dependencies, making it particularly effective in processing images with complex semantic structures. Additionally, since ViT lacks the inherent translational invariance of convolutional networks, it employs positional encoding to preserve the spatial location information of image blocks. This positional encoding ensures that the model accurately captures the spatial structure of the image by embedding positional information within each image block feature.

For the input image *I*, ViT first segments it into fixed-size image blocks $P=[p_{1},p_{2},\ldots ,p_{n_{v}}]$, and each image block *p*_*i*_ is linearly projected into a high-dimensional space with an additional positional encoding to preserve the spatial information, as shown in Eq (2):

$$\begin{array}{c} p_{i}^{v}=W_{p}\cdot \text{vec}(p_{i})+{\text{pos}}_{i} \end{array}$$ (2)

where, **W**_*p*_ is the learnable linear projection matrix, vec(*p*_*i*_) is the vectorized representation of the image block, and pos_*i*_ is the positional encoding.

After obtaining the serialized image blocks, ViT applies the Multi-Head Self-Attention (MSA) mechanism to process these image blocks so that the model can learn the dependencies between the blocks as shown in Eqs (3) to (4):

$$\begin{array}{c} Z^{\left(0\right)}=[p_{1}^{v},p_{2}^{v},\ldots ,p_{n_{p}}^{v}] \end{array}$$ (3)

$$\begin{array}{c} Z^{\left(I\right)}=\text{MSA}(\text{LN}(Z^{\left(I-1\right)}))+Z^{\left(I-1\right)},\text{for}I=1\ldots L \end{array}$$ (4)

where, *n*_*v*_ is the number of image blocks, **Z**^(0)^ is the sequence of image blocks after projection, **Z**^(*I*)^ is the output of the *I*th layer, denotes Layer Normalization, and *L* is the number of layers of Transformer. The parallel processing capability of the multi-head self-attention mechanism enables the model to interpret image content from multiple perspectives, thereby enhancing its ability to capture complex image semantics. In news images, different attention heads can focus on various regions of the image, such as text areas, facial features, and key objects, thus acquiring more comprehensive semantic information. After multiple layers of self-attentive processing, the final output **Z**^(*L*)^ is passed to a Feed-Forward Neural Network (FFN) to obtain the final representation of each image block as shown in Eq (5):

$$\begin{array}{c} Z_{final}=\text{FFN}(\text{LN}(Z^{\left(L\right)})) \end{array}$$ (5)

where, the output of the ViT module *Z*_*final*_ represents the rich semantic information of the image and is suitable for subsequent tasks.

### Visual tampering branch

The primary objective of the visual tampering branch is to detect traces of tampering in news images and identify regions that have been maliciously modified or inserted. The Enhanced Vision Transformer (EAViT) proposed by J. Horváth et al. [49] is a specially designed model for image tampering detection, which is optimized on the basis of the traditional vision transformer (ViT) to extract and identify tampered image regions. In the algorithm of this paper, EAViT is used to identify objects or regions that may have been maliciously modified or inserted into the original image. Each image block is converted to an image token **T**_*i*_ by a linear mapping function W, as shown in Eq (6):

$$\begin{array}{c} T_{i}=\text{W}\left(p_{i}\right) \end{array}$$ (6)

where, W is a learnable linear projection matrix and *p*_*i*_ is a fixed size image block. These image tokens are input as sequences into the EAViT model.

The EAViT model is extended by adding an autoencoder structure to ViT that allows it to learn and reconstruct the distribution of an untampered image. In the testing phase, given an image *I* that may contain tampered regions, EAViT will attempt to reconstruct the image using the distribution of the untampered image that it learned during training to obtain the reconstructed image *I*_*r*_. The following smoothing L1 loss function is utilized to optimize the model parameters to minimize the difference between the input image and the reconstructed image as shown in Eq (7):

$$\begin{array}{c} {\text{L}}_{r}(I,I_{r})=\frac{1}{\left|I\right|}\sum_{i}\{\begin{array}{cc} \frac{1}{2}{\left(I\left(i\right)-I_{r}\left(i\right)\right)}^{2}, & \text{if}|I\left(i\right)-I_{r}\left(i\right)|<1\\ |I\left(i\right)-I_{r}\left(i\right)|-\frac{1}{2}, & \text{otherwise} \end{array} \end{array}$$ (7)

The loss function is designed to minimize the pixel differences between the input image and the reconstructed image, thereby helping the model learn the true distribution of untampered images. The discrepancies between the reconstructed image and the input image tend to be concentrated in the tampered regions, allowing these differences to be used to identify traces of tampering.

Next, the Laplace filter *f*_*L*_ of 3 × 3 is applied to the input image *I* and the reconstructed image *I*_*r*_ respectively to generate two new images *I*_*d*_ and *I*_*rd*_ as shown in Eqs (8) to (9):

$$\begin{array}{c} I_{d}=I*f_{L} \end{array}$$ (8)

$$\begin{array}{c} I_{rd}=I_{r}*f_{L} \end{array}$$ (9)

The purpose of utilizing the Laplace filter is because autoencoders may have difficulty in reconstructing the high frequency components of an image, and the Laplace filter as an edge detector can highlight these high frequency components. Heat maps are constructed by averaging the difference between *I* and *I*_*r*_, *I*_*d*_ and *I*_*rd*_, showing the potential tampered regions within the image. Subsequently, the heat map is thresholded to create a binary mask and a morphological filter is used to output the mask that ultimately indicates the tampered region in the post-processing stage.

### Co-attention network

This subsection proposes multimodal-aware co-attention networks with mutual knowledge refinement, aimed at enhancing the performance of fake news detection tasks by capturing cross-modal relationships between images and text. The module designs a new multimodal-aware co-attention network to capture matching information of images and texts in order to learn multimodal representations. Based on the new co-attention mechanism, the collaborative attention network consists of two main components: a text-centered attention network and an image-centered attention network. These networks enable mutual knowledge refinement, thereby improving fake news detection. Multimodal features are fused through two multimodal-aware joint attention networks, which are centered on text (textual features as query) and image (image features as query), respectively. After that, the output of the co-attention network to MMoE is used for fake news classification. Mutual learning is used between the two co-attention networks for mutual knowledge refinement to synergistically improve fake news detection.

A pre-trained BERT model is used to extract feature vectors from news text **T**, while Visual Transformer (ViT) is used to extract visual feature vectors from related images **I**. These two types of feature extraction ensure that rich semantic information is captured from each modality.

The implementation of the co-attention mechanism focuses on processing these features through a multi-head attention model. In this model, the textual feature **T** is converted into a Query, while the image feature **I** acts as both Key and Value, thus realizing the flow of information from text to image. This is shown in Eq (10):

$$\begin{array}{c} Q=TW^{Q},K=IW^{K},V=IW^{V} \end{array}$$ (10)

where, **W**^**Q**^, **W**^**K**^, **W**^**V**^ are learnable transformation matrices. The computation of multiple attention is accomplished through Eq (11):

$$\begin{array}{c} \text{Attention}\left(Q,K,V\right)=\text{softmax}(\frac{QK^{T}}{\sqrt{d_{k}}})V \end{array}$$ (11)

where, *d*_*k*_ denotes the dimension of the key vector, this scaling factor helps to stabilize the gradient during training.

The weighted image features computed by the above attention mechanism are combined with the original textual features to form a fusion feature **F**, which is enhanced by the ReLU activation function and further linear transformation as shown in Eq (12):

$$\begin{array}{c} F=\text{ReLU}(TW^{T}+\text{Attention}(TW^{Q},IW^{K},IW^{V})W^{F}) \end{array}$$ (12)

where, **W**^**T**^ and **W**^**F**^ are learnable weight matrices for further tuning the feature representation.

### Cross-modal feature branch

The primary objective of the cross-modal feature branch is to leverage cross-modal learning techniques to establish connections between images and text and extract joint feature representations. The CLIP (Contrastive Language-Image Pretraining) model, a multimodal pre-training model, captures the rich semantic relationships between images and text by learning their representations simultaneously. This model effectively captures the semantic consistency between textual descriptions and image content, aiding in the detection of fake news. The cross-modal feature branch utilizes two key components of the CLIP model: the image encoder and the text encoder. These encoders convert the input images and text into feature vectors within a unified representation space, and the similarity between these vectors is calculated to assess the relevance between the image and text.

The image encoder receives the input image *I*, which is converted into an image feature vector **I**^*c*^ by the pre-trained CLIP model as shown in Eq (13):

$$\begin{array}{c} I^{c}=\;{\text{CLIP}}_{\text{ImageEncoder}}\left(I\right) \end{array}$$ (13)

the text encoder receives the news text *T*, which is converted into a textual feature vector **T**^*c*^ as shown in Eq (14):

$$\begin{array}{c} T^{c}=\;{\text{CLIP}}_{\text{Text}\;\text{Encoder}}\left(T\right) \end{array}$$ (14)

both the image and text encoders built into the CLIP model use the Transformer architecture. The image encoder splits the image into chunks and treats each chunk as a sequence element and extracts image features using a self-attention mechanism. The text encoder, on the other hand, splits the input text into words and encodes them as textual features using the same self-attention mechanism.

The image features **I**^*c*^ and textual features **T**^*c*^ extracted by the CLIP encoder will be used to evaluate the consistency between the image content and the text description by means of a similarity metric. This metric can be shown in Eq (15):

$$\begin{array}{c} S(I^{c},T^{c})=\frac{I^{c}\cdot T^{c}}{∥I^{c}∥\cdot ∥T^{c}∥} \end{array}$$ (15)

where, *S* is the cosine similarity between the image and textual feature vectors, which is used to determine whether the two modalities match each other. Higher similarity scores indicate that the image content and text description are highly consistent, while lower scores may imply that there is false information in the news content. By calculating the similarity between each image-text pair, the cross-modal feature branch generates a cross-modal similarity matrix for subsequent classification tasks.Additionally, the image and text feature vectors **I**^*c*^ and **T**^*c*^ generated by CLIP are directly used for further feature fusion, resulting in a more robust multimodal feature representation.

### Large language model branch

Large Language Models (LLMs), such as GPT-4, provide advanced text generation capabilities that can be used to generate explanatory justifications for fake news detection. This helps to extract subtle text patterns and enhance the interpretability of machine learning predictions. In this paper, we take an adaptive method to utilize the insights of LLMs to guide SLMs and improve their performance. The method utilizes the deep background knowledge and multi-perspective reasoning provided by LLMs, especially GPT-4, to enhance the judgment of BERT. The system utilizes the task-specific learning capabilities of BERT while incorporating the broad insights of GPT-4.

**Fig 2:**
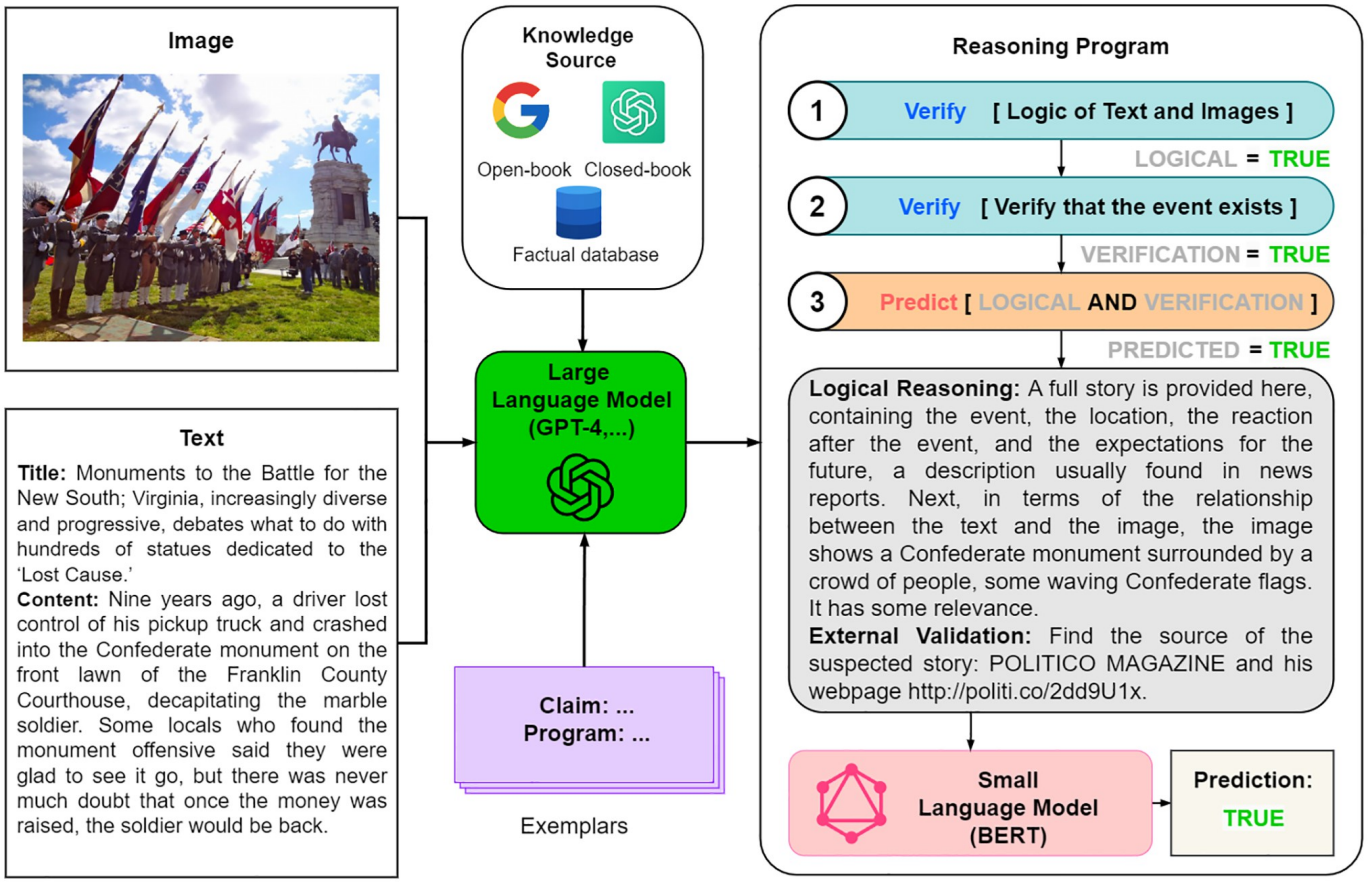
Overview of the large language model branch.

Fig 2 illustrates the processing of the large language model branch, which utilizes large language models, such as Codex or GPT-4, to validate news statements from multiple knowledge sources. The consistency of text and images is analyzed through two steps, logical reasoning and external validation, to check whether the reported events are true or not. The results of the two steps are combined to predict the truthfulness of the news by means of a small language model (e.g., BERT). The process emphasizes the logical relationship between text and images and the verification of external knowledge, thus ensuring the comprehensiveness and depth of the information review.

For a given news article *X*, LLMs are used to generate the rationale *R*, which provides a rich perspective context for determining the authenticity of *X*. The set of justifications can be represented as *R* = [*r*_1_, *r*_2_, …, *r*_*n*_], and for each justification *r*_*i*_, BERT processes and encodes it as an embedding **e**_*i*_, as shown in Eq (16):

$$\begin{array}{c} e_{i}={\text{BERT}}_{Encoder}\left(r_{i}\right) \end{array}$$ (16)

where, the embedding **e**_*i*_ is integrated by weighting to form the comprehensive insight vector *E* which encapsulates the collective knowledge of the rationale as shown in Eq (17):

$$\begin{array}{c} E=\sum_{i=1}^{n}w_{i}\cdot e_{i} \end{array}$$ (17)

where, the weights *w*_*i*_ are obtained through a trainable attention mechanism that takes into account the content of the news article and the associated rationale as shown in Eq (18):

$$\begin{array}{c} w_{i}=\text{Attention}(e_{i},H_{x}) \end{array}$$ (18)

where, **H**_*x*_ is the hidden representation of the article *X*, encoded by BERT. BERT is utilized to parse the logical relationships between sentences and paragraphs in the news text and calculate the consistency score for each logical unit as shown in Eq (19):

$$\begin{array}{c} L\left(u\right)=\text{sigmoid}({\text{BERT}}_{logic}\left(u\right)) \end{array}$$ (19)

where, *u* denotes logical units in the text and BERT_*logic*_ is a variant of the BERT model specifically trained to assess the logical consistency of text. This method is able to assess whether the language in news reports is logically self-consistent, and whether there are any logical contradictions or faulty reasoning.

In addition to logical reasoning, external validation is an important feature of the module, which extracts key facts from trusted data sources using a pre-trained BERT model to form a fact database F. For each news item, key statements are extracted from the news text and BERT is used to evaluate the consistency of these statements with the facts in the fact database. The truthfulness of the news content can be quantified by calculating a similarity score between each statement and fact. The similarity score is calculated by Eq (20):

$$\begin{array}{c} \text{score}\left(s,f\right)=\frac{\text{exp}({\text{BERT}}_{sim}\left(s,f\right))}{\sum_{f^{\prime }\in F}\text{exp}({\text{BERT}}_{sim}(s,f^{\prime }))} \end{array}$$ (20)

where, *s* denotes statements in the news and *f* and *f*′ denote facts in the fact database. This methodology ensures that it is possible to quantify and assess the consistency of news content with recognized facts.

The features obtained from logical inference and external validation are fused to perform the final classification of fake news using a multilayer perceptron (MLP). The fused features are shown in Eq (21):

$$\begin{array}{c} F_{total}=\text{Concat}(F_{fact},F_{logic}) \end{array}$$ (21)

where, **F**_*fact*_ and **F**_*logic*_ represent the feature vectors obtained from logical reasoning and external verification, respectively. In this way, the algorithm in this paper can not only accurately determine the truthfulness of the news, but also further verify the reasonableness of the news content through logical reasoning:

Finally, the enhanced feature representation **F** of the article *X* is constituted by combining its encoded form **H**_*x*_ with the integrated rationale insight **E** as shown in Eq (22):

$$\begin{array}{c} F=a\cdot H_{x}+\left(1-a\right)\cdot E \end{array}$$ (22)

where, *a* is a hyperparameter that balances the contribution of the original article content with the rationale provided by LLMs.

With this integration, the fine-tuned BERT benefits from the advanced insights provided by LLMs, making the detection of fake news smarter and more accurate.

### Multidimensional feature integration network

The primary function of the Multidimensional Feature Integration Network (MMoE) is to integrate feature representations from different modalities and fuse these features to enhance the overall performance of the fake news detection model. In fake news detection tasks, features from various modalities may capture different types of information. By integrating this information, a more comprehensive feature representation can be obtained, thereby improving the model’s classification accuracy. The MMoE module is designed to integrate features from different modalities and make decisions for the fake news detection task. The module utilizes multiple Expert Networks and a Gating Network to achieve feature integration. Each Expert Network focuses on learning specific features from different modalities, while the Gating Network contributes to the final prediction by adjusting the membership weights based on the characteristics of the input data, ensuring optimal feature fusion.

For a given news article *A*, there are textual features **T**^*c*^, image features **I**^*c*^, and features output from the ground truth encoder **E**_*i*_. MMoE processes these features through a series of expert networks Expert_*k*_ and computes the corresponding gating weights *g*_*k*_ for each expert network as shown in Eqs (23) to (24):

$$\begin{array}{c} E_{k}={\text{Expert}}_{k}\left(T^{c},I^{c},E_{i}\right) \end{array}$$ (23)

$$\begin{array}{c} g_{k}={\text{Gate}}_{k}\left(T^{c},I^{c},E_{i}\right) \end{array}$$ (24)

where, *k* denotes the first *k* expert network. The gating mechanism ensures that the sum of all gating weights is 1 through the softmax function as shown in Eq (25):

$$\begin{array}{c} \sum_{k}g_{k}=1,\text{where}g_{k}=\frac{\text{exp}({\text{GateNet}}_{k}\left(T^{c},I^{c},E_{i}\right))}{\sum_{j}\text{exp}({\text{GateNet}}_{j}\left(T^{c},I^{c},E_{i}\right))} \end{array}$$ (25)

then, the output of each expert is integrated based on the gating weights to form the final predicted features **F**, as shown in Eq (26):

$$\begin{array}{c} F=\sum_{k}g_{k}\cdot E_{k} \end{array}$$ (26)

This feature representation integrates comprehensive information from different modalities and is optimized through the Expert Networks and Gating Network, resulting in enhanced expressiveness and discriminative power. The final fused features are then fed into a classifier for fake news detection and classification. In the task of fake news detection, the features of different news items may exhibit high heterogeneity. The MMoE framework, through its Expert Networks and gating mechanism, effectively captures this heterogeneity and provides highly discriminative feature representations, thereby improving the model’s performance in detecting fake news in complex scenarios.

### The final classifier

The final classifier is responsible for mapping the features **F** integrated by the MMoE module to the authenticity labels of the news. This mapping is realized by a classifier, usually a multilayer perceptron (MLP). This MLP contains one or more fully connected layers and includes nonlinear activation functions such as ReLU or tanh to increase the nonlinear expressiveness of the model. The last layer is the softmax layer, which converts the output into predicted probabilities for each category.

Here output of the classifier is denoted as $\hat{y}$, as shown in Eq (27):

$$\begin{array}{c} \hat{y}=\text{softmax}\left(\text{MLP}\left(F\right)\right) \end{array}$$ (27)

where, the softmax function is defined as shown in Eq (28):

$$\begin{array}{c} \text{softmax}\left(z_{i}\right)=\frac{\text{exp}(z_{i})}{\sum_{j=1}^{M}\text{exp}(z_{j})} \end{array}$$ (28)

for each sample *i*, *z*_*i*_ is the raw log odds (logits) of the output of the last layer of the MLP and $\hat{y}$ is the probability distribution predicted by the model. During training, the model’s objective is to minimize the error between the predicted results and the actual labels.

In order to train the model and minimize the error between the predicted label ${\hat{y}}_{\text{i}}$ and the true label *y*, a cross-entropy loss function is evaluated. Given a sample with the true label $\hat{y}$ (in the dichotomous case, $\hat{y}$ is either 0 or 1) and the corresponding predicted probability ${\hat{y}}_{\text{i}}$, the cross-entropy loss is defined as shown in Eq (29):

$$\begin{array}{c} L=-\frac{1}{N}\sum_{i=1}^{N}[y_{i}\;\text{log}\;({\hat{y}}_{i})+(1-y_{i})\;\text{log}\;(1-{\hat{y}}_{i})] \end{array}$$ (29)

where, *N* is the total sample size. The loss function is optimized over the entire training dataset to adjust the weights in the MLP to improve the model’s prediction accuracy for fake news. After training is complete, the final classifier inputs the fused multimodal features **F** into the MLP to generate a probability distribution $\hat{y}$ . By selecting the label with the highest probability, the model arrives at the final classification result, determining the authenticity of the news content.

## Experimentation and analysis

### Dataset

This paper evaluates the performance of fake news detection models using three datasets collected from real social media platforms: Weibo, Gossipcop and Politifact. The detailed statistical information of the datasets is shown in Table 2.

**Table 2:** Dataset statistics.

Dataset	Tab	Quantities	Aggregate
Weibo	Real news	4779	9528
	Fake news	4749	
GossipCop	Real news	16817	22140
	Fake news	5323	
PolitiFact	Real news	624	1056
	Fake news	432	

Weibo [33] is the first Chinese fake news dataset constructed on the Sina Weibo platform by Jin et al. The dataset covers fake news verified by Weibo’s official disinformation platform from May 2012 to January 2016, as well as real news verified by Xinhua News Agency during the same period. In the Weibo dataset, 80% of the dataset is randomly selected as the training set 80%, and the remaining 20% is used as the test set.

FakeNewsNet [50] comes from Twitter, the most popular social media platform in the U.S. It contains two datasets: PolitiFact and GossipCop. PolitiFact is a prominent nonprofit political statement and website that reports on fact-checking in the U.S. The PolitiFact dataset consists of news related to U.S. politics. The GossipCop dataset is collected from FakeNewsNet’s Entertainment domain in English long form, focusing on news related to Hollywood celebrities. dataset is a collection of English-language long-form articles from FakeNewsNet’s Entertainment domain that focuses on news related to Hollywood celebrities. For the PolitiFact and GossipCop datasets, this paper divides the training, validation, and test sets in the ratio of 7:1:2.

### Experimental setup

In this paper, Accuracy, Precision, Recall and F1-score are used as the evaluation metrics for model detection performance. The experiments are all based on Python3.10 and PyTorch1.13 environments, using NVIDIA A100 80GB GPU. after enabling mixed-precision training, the video memory usage is reduced by about 50% and stabilized between 25GB and 40GB. The specific experimental parameters are shown in Table 3.

**Table 3:** Experimental parameter settings.

Parameters	Value
Epoch	1000
Learning rate	0.001
Weight decay	0.01
Dropout	0.4
BatchSize	32
Optimizer	Adam

### Results

In order to verify the effectiveness of the proposed method in the task of fake news detection, this paper compares the performance of various types of baseline methods for fake news detection.

BERT [11]: Textual features are extracted using a pre-trained BERT model and classified using a fully connected layer.

VGG19 [51]: News visual features are extracted using a VGG pre-trained model and classified using a fully connected layer.

att-RNN [33]: The framework utilizes a Long Short-Term Memory Network (LSTM) to extract textual and social contextual features, combined with visual features extracted by a VGG pre-trained model and cross-modal feature fusion through the attention mechanism, and finally feeds the fused features into a classifier for the recognition of fake news.

EANN [35]: Detecting fake news through multimodal feature extraction as well as event-based adversarial networks. The model consists of three main parts: a multimodal feature extractor, a fake news detector, and an event discriminator. Text and image features are extracted using TextCNN and VGG pre-trained models, respectively, and these features are spliced and input into the fake news detector. To ensure the fairness of the comparison, the event discriminator part is removed in this paper.

MVAE [36]: Textual and visual features are extracted using bi-directional LSTM model and pre-trained VGG model respectively, multimodal features are obtained by concatenating them, and then the multimodal feature distribution is learned using variational self-encoder.

SAFE [39]: Multimodal Fake News Detection by Similarity Analysis. An LSTM temporal model and a VGG pre-trained model are used to extract textual and visual features of the news respectively, and then the semantic similarity between these features is computed to analyze the cross-modal semantic associations of the news, and then predict the authenticity of the news.

Spotfake+ [52]: Extracts textual and visual features of news using XLNet and VGG pre-trained models, and concatenates these features for fake news detection.

LSTM-ATT [53]: builds an XGBoost-based model to detect whole fake news.

DistilBert [54]: Uses correlation between user-generated content and user-shared content to detect fake news.

CAFE [15]: Adaptive aggregation of unimodal features of text and images as well as cross-modal correlations to detect fake news through cross-modal manifold learning. Textual and visual features are extracted using TextCNN and VGG pre-trained models, and the two types of features are spliced and input to a news classifier for classification.

CMC [14]: Multimodal Fake News Detection Using Correlation of Cross-Modal Features through a Novel Method to Knowledge Refinement.

The experimental results of the proposed method and baseline model in this paper on three representative datasets are shown in Table 4. The following conclusions can be drawn from the experimental results in Table 3:

**Table 4:** Comparison of experimental results.

Dataset	Method	Accuracy	Fake news			Real news		
			Precision	Recall	F1-score	Precision	Recall	F1-score
Weibo	BERT	0.804	0.798	0.831	0.814	0.811	0.776	0.793
	VGG-19	0.635	0.63	0.706	0.666	0.641	0.559	0.597
	att-RNN	0.772	0.854	0.656	0.742	0.72	0.889	0.795
	EANN	0.827	0.847	0.812	0.829	0.807	0.843	0.825
	MVAE	0.824	0.854	0.769	0.809	0.802	0.875	0.837
	SAFE	0.763	0.833	0.659	0.736	0.717	0.868	0.758
	Spotfake+	0.87	0.887	0.849	0.868	0.855	0.892	0.873
	CAFE	0.84	0.855	0.83	0.842	0.825	0.851	0.837
	CMC	0.908	0.94	0.869	0.899	0.876	0.945	0.907
	Ours	0.912	0.915	0.911	0.913	0.908	0.913	0.91
GossipCop	att-RNN	0.743	-	-	-	0.788	0.913	0.846
	DistilBert	0.857	0.805	0.527	0.637	0.866	0.96	0.911
	SAFE	0.838	0.758	0.558	0.643	0.857	0.937	0.895
	Spotfake+	0.856	-	-	-	-	-	-
	LSTM-ATT	0.842	0.845	0.842	0.844	0.839	0.842	0.821
	CAFE	0.867	0.732	0.49	0.587	0.887	0.957	0.921
	CMC	0.893	0.826	0.657	0.692	0.92	0.963	0.935
	Ours	0.905	0.85	0.712	0.775	0.922	0.962	0.942
PolitiFact	att-RNN	0.769	-	-	-	0.735	0.942	0.826
	DistilBert	0.741	0.875	0.636	0.737	0.647	0.88	0.746
	SAFE	0.874	0.851	0.83	0.84	0.889	0.903	0.896
	Spotfake+	0.846	-	-	-	-	-	-
	LSTM-ATT	0.832	0.828	0.832	0.83	0.836	0.832	0.829
	CAFE	0.864	0.724	0.778	0.75	0.895	0.919	0.907
	CMC	0.894	0.806	0.862	0.833	0.944	0.92	0.932
	Ours	0.926	0.882	0.905	0.893	0.95	0.937	0.943

- In the unimodal model, the text-based detection method outperforms the image-based detection method, which reflects the strong dependence on textual information for fake news detection and the inadequacy of analyzing visual semantic cues. On the other hand, the fake news detection model incorporating multimodal information shows advantages over unimodal methods, confirming the importance of the complementary nature of textual and visual information in improving detection performance.
- In the multimodal model, DistilBert detects fake news by correlating article and user information without fully exploiting visual information. EANN and Att-RNN only rely on direct splicing or cross-modal attention mechanisms to obtain fused features, which cannot provide sufficient discriminative power for fake news classification. The global metrics utilized by SAFE are unable to efficiently capture cross-modal semantic interactions of news and lacks in-depth insight into the intrinsic characteristics of multimodal news. Spotfake+ simply concatenates textual and visual representations without sufficient cross-modal interactions and fusion, resulting in unsatisfactory performance. CMC adopts a two-phase cross-modal knowledge distillation method to make full use of the relevance of the cross-modal features, and thus its accuracy is better than that of the other models.
- The accuracy of the method proposed in this paper outperforms other methods on the three datasets of Weibo, Gossipcop, and Politifact, reaching 91.2%, 90.5%, and 92.6%, respectively, and exceeding the best results of the compared methods by 0.4%, 1.2%, and 3.2%. In terms of precision, recall and F1-score, the method proposed in this paper ranks first or second in almost all the tests, which indicates that the method proposed in this paper can effectively improve the performance of fake news detection while having the ability to generalize across different datasets.

### Ablation experiment

In order to verify the validity of each component in the model, five variants of the model are designed in this paper to perform ablation analysis of the model, and the results are shown in Table 5.

- Text: only unimodal textual features extracted by BERT are used for detection.
- Image: detection using only unimodal visual features extracted by ViT.
- FND-LLM-C: Remove all branches related to CLIP and keep only BERT and ViT to extract textual and visual features.
- FND-LLM-A: Remove joint attention branch and directly use unimodal textual and visual features.
- FND-LLM-L: Remove the large language model branch and rely only on textual and visual features for detection.

**Table 5:** Ablation experiments.

Dataset	Method	Accuracy	Precision	Recall	F1-score
Weibo	Text	0.871	0.831	0.883	0.856
	Image	0.789	0.776	0.865	0.818
	FND-LLM-C	0.883	0.863	0.915	0.888
	FND-LLM-A	0.898	0.866	0.941	0.902
	FND-LLM-L	0.896	0.866	0.924	0.894
	FND-LLM	0.912	0.908	0.913	0.91
GossipCop	Text	0.871	0.889	0.936	0.912
	Image	0.736	0.763	0.841	0.8
	FND-LLM-C	0.881	0.869	0.951	0.908
	FND-LLM-A	0.9	0.905	0.931	0.918
	FND-LLM-L	0.893	0.922	0.949	0.935
	FND-LLM	0.905	0.922	0.962	0.942
PolitiFact	Text	0.882	0.898	0.939	0.918
	Image	0.696	0.699	0.902	0.788
	FND-LLM-C	0.909	0.913	0.91	0.912
	FND-LLM-A	0.926	0.929	0.971	0.95
	FND-LLM-L	0.901	0.909	0.942	0.925
	FND-LLM	0.926	0.95	0.937	0.943

Table 5 lists the experimental results of removing each important module or method of the model for ablation analysis, and the following conclusions are observed.

- Removing any important part or important method of the model results in varying degrees of degradation in model performance, which demonstrates the effectiveness of the modules in the model proposed in this paper in the multimodal fake news detection task.
- According to the degree of decline in accuracy, only retaining the visually relevant branch Image model performance of the most obvious degree of decline in the three datasets, Text’s performance is much better than Image’s performance, indicating that the textual content is more important in the detection of fake news, the visual information can only be used as a complementary feature, is not enough to carry out classification.
- FND-LLM outperforms FND-LLM-L, indicating that the large language model has the potential for fake news detection, and can provide reasonable and informative justifications through logical reasoning and external validation to make up for the limitations of the small language model, improving the model performance while enhancing the interpretability of the detection process.
- FND-LLM outperforms FND-LLM-C, demonstrating that the cross-modal features provided by CLIP help to improve fake news classification accuracy. Although fake news detection can be performed by relying on intra-modal features only, the final features cannot reflect the intrinsic relationship between images and text due to the lack of inter-modal interaction.
- FND-LLM outperforms FND-LLM-A on the Weibo and Gossipcop datasets, suggesting that the multimodal attention network can help FND-LLM adaptively weight useful modalities, and that FND-LLM-A directly fuses the features of different modalities, which may result in the final features being affected by the invalid information in some of the modalities.

## Conclusion

Addressing the limitations of small language models in dealing with specialized knowledge and the under-exploited potential of LLMs in fake news detection, this paper proposes a LLM-enhanced multimodal fake news detection method. After extensive iterations, the LLMs have developed robust multimodal processing capabilities and complex reasoning abilities. Consequently, this paper leverages the LLMs to generate multi-angle guiding opinions through logical reasoning and external validation of news content, effectively complementing the capabilities of SLMs. Driven by both unimodal prediction and cross-modal consistency learning methods, we meticulously differentiate and optimize the information content in unimodal and multimodal features through adaptive weight allocation and guidance mechanisms, achieving more effective detection results. Comprehensive experiments conducted on three well-known datasets show that our method outperforms many advanced methods in terms of accuracy.

Looking ahead, we plan to implement a “Dynamic Knowledge Prompt Enhancement” approach to continuously improve the performance of our multimodal fake news detection model. This method aims to boost the effectiveness of large language models (LLMs) by incorporating domain-specific terminology, relationships, and contextual information, thereby improving task execution, adaptability, and scalability.

As technology advances and research progresses, “Dynamic Knowledge Prompt Enhancement” is expected to become a long-term, effective strategy that not only significantly improves fake news detection accuracy but also offers valuable insights for other domains.

## Supporting information

S1 Data Minimal data set. (CSV)
